# More Data and Appropriate Statistical Methods Needed to Fully Measure the Displacement Effects of Development Assistance for Health

**DOI:** 10.1371/journal.pmed.1001365

**Published:** 2013-01-08

**Authors:** Christopher J. L. Murray, Joseph L. Dieleman, Chunling Lu, Michael Hanlon

**Affiliations:** 1 Institute for Health Metrics and Evaluation, University of Washington, Seattle, Washington, United States of America; 2 Department of Global Health and Social Medicine, Harvard Medical School, Boston, Massachusetts, United States of America

## Abstract

Christopher Murray and colleagues provide a perspective on displacement due to development assistance for health, and argue for more data and better statistical methods to measure aid displacement.

**Linked Perspective Articles**

Batniji R, Bendavid E (2013) Considerations in Assessing the Evidence and Implications of Aid Displacement from the Health Sector. PLoS Med 10(1): e1001364. doi:10.1371/journal.pmed.1001364

Murray CJL, Dieleman JL, Lu C, Hanlon M (2013) More Data and Appropriate Statistical Methods Needed to Fully Measure the Displacement Effects of Development Assistance for Health. PLoS Med 10(1): e1001365. doi:10.1371/journal.pmed.1001365

The question of whether foreign aid results in recipient governments spending less on health is an enduring concern in global health. In separate analyses, Farag et al. and Lu et al., which included members of our group (CL, CM), found compelling evidence of “aid displacement," meaning a substitution between development assistance for health from foreign donors and health expenditure financed by domestic sources [1],[2] We reported that for every dollar a government received in development assistance for health, the government's domestic expenditure on health decreased by $0.46 (95% confidence interval: 0.24, 0.67) [2]. Subsequent research questioned those findings [3]–[6], including Batniji and Bendavid in *PLOS Medicine* who have since admitted mistakes in their statistical model [4]. We argue that these subsequent studies fail to account for the complex, dynamic connection between domestic and foreign funding sources. Estimating this relationship requires detailed data on both flows of resources and how national resources are allocated along with the appropriate statistical method. Given the analytical challenges, it is imperative that more data be available and appropriate methods be used, so that the impact of development assistance can be better understood.

## Future Research

Methodologists will continue to debate how best to model this relationship, but the prevailing evidence is clear that on average displacement is occurring, and, at a minimum, reductions in government health expenditure are significantly associated with increases in development assistance channeled to the government. The next challenges are to (a) further substantiate this effect and (b) unpack the average effect so that we better understand the subtle but important characteristics of this behavior. Important research questions should answer when and where aid displacement is greatest. Current models presume countries to be financially stationary by assuming that all the non-modeled time-variant effects do not systematically affect government health expenditure. This may not be the case. Furthermore, the degree of displacement may vary across several dimensions. Future analyses should consider if the rate of displacement changes over time or across health financing scheme or aid focus areas. Finally, the rate of replacement caused by aid decreases might not equal the rate of displacement caused by aid increases. In other words, it is unknown if the displacement effect is symmetric. The answers to all these questions would dramatically influence the long-term impact of development assistance for health.

In the long run, it is unclear how displacement between development assistance and government spending affects societal welfare. For example, funds may be reallocated from health to the education sector, which also has the potential to generate social value. Due to limited data, we have no meaningful knowledge of how displaced funds are reallocated and how much social value is generated by alternate activities. Since government resources are often not ear-marked, displacement in the health sector may have non-trivial effects on a host of sectors. This ambiguity highlights the need for better data on both development assistance and government expenditure across sectors.

## What Data Exist, and What Data Are Needed

Data from three financial flows can significantly improve our understanding of aid displacement: (i) development assistance, disaggregated by sector (health, education, et cetera) and recipient agent (government or non-government); (ii) government expenditure, disaggregated by sector; and (iii) governmental tax revenues. To thoroughly address this question, these data series would be required at the country-year level. If these data existed and expected resources could be tracked across sectors, then researchers, donors, and policy makers would have much clearer insight on the displacement of various funding streams, and begin to explore the effect of that behavior on population welfare.

As a practical matter, some elements of each of these series are currently unavailable. The Institute for Health Metrics and Evaluation (IHME) regularly publishes policy reports on development assistance or health [7]–[10]. The underlying data for these reports are disaggregated by funding sources, channels of assistance, implementing institutions, recipient countries, and health focus areas, and are publically available at the IHME Global Health Data Exchange [11]. The Development Assistance Committee (DAC) of the Organisation for Economic Co-operation and Development maintains the Creditor Reporting System (CRS), which reports project-level information about official development assistance, other official flows, and some private assistance [12]. The CRS provides most—but not all—of the information necessary to estimate the total amount of development assistance across all sectors. To supplement the CRS, researchers would need to solicit data from bilateral donors, multilateral agencies, and relevant non-governmental organizations that do not actively participate in the DAC. This would be an intensive process, especially to disaggregate the total by sector, but it is akin to how IHME generates its series for development assistance to health.

Government health expenditure is reported by the World Health Organization (WHO), although that estimate comingles domestically generated funds with development assistance for health channeled through the government [13]. By subtracting the amount of development assistance channeled to the government in a given year, we can estimate a first approximation of governmental health expenditure from domestically generated sources. The International Monetary Fund (IMF) reports for some countries on total government expenditures [14]. The main challenge is that the IMF Government Finance Statistics provide a breakdown of government spending by sector for very few countries. Despite the urgency of understanding how development assistance affects government spending patterns, improvements in tracking government spending by sector are not currently a priority.

Even tracking total government tax revenues is a challenge. The most reliable sources of revenue data are from IMF surveillance documents and in particular the Article IV consultation reports [15]. From 1998 to 2012, a total of 1,581 reports from 188 countries are publicly available (which implies 40% of this series' country years are either unavailable or missing). One strategy to complete these series would be to use a variety of data sources, such as national account matrices and household budget surveys, to triangulate on an estimate of the true value. The statistical techniques to accomplish this estimation are well established (a recent example is estimating the ownership of insecticide-treated bed nets [16]). However, the task of compiling the complementary data is not trivial, and it would require the commitment of funding from donors interested in thoroughly addressing the issue of how displacement affects social welfare.

On average, the evidence is that a tradeoff exists between development assistance channeled to the government health sector and health expenditure financed by domestic sources. To better understand this phenomenon, documentation of how governments spend their own resources by sector is an urgent priority. Improved data, which is in the interest of governments, donors, and concerned citizens alike, will need a concerted effort by the development community. Further improvements in data and the appropriate statistical methods should lead to more rigorous and detailed analyses of the total impact of development assistance.

## Abbreviations

CRS: Creditor Reporting System
DAC: Development Assistance Committee
IHME: Institute for Health Metrics and Evaluation
IMF: International Monetary Fund
WHO: World Health Organization
